# ADVANCE CARE PLANNING IN DEMENTIA VERSUS NORMAL COGNITION: A DEMOGRAPHIC AND PSYCHOSOCIAL STUDY

**DOI:** 10.1093/geroni/igae098.1301

**Published:** 2024-12-31

**Authors:** Zahra Rahemi, Juanita Bacsu, Swann Adams

**Affiliations:** Clemson University, Clemson, South Carolina, United States; Thompson Rivers University, Kamloops, BC Canada, British Columbia, Canada; University of South Carolina, Columbia, South Carolina, United States

## Abstract

Older adults from minority groups often face higher rates of chronic diseases and cognitive impairment, along with lower rates of advance care planning and comfort care at end-of-life. This study aimed to explore patterns of advance care planning among older adults in the U.S., focusing on various demographic and psychosocial factors and cognition levels. We utilized data from the 2018 Health and Retirement Study to investigate the association between these factors and the likelihood of having a living will, a durable power of attorney for healthcare (DPOAH), or both, stratified by cognition (dementia/impaired cognition vs. normal cognition). Variables such as race, ethnicity, rurality, marital status, gender, education, age, and discrimination were included in the models. Among the 17,698 respondents, 71.6% exhibited normal cognition, while 28.4% had been diagnosed with dementia or impaired cognition. In both cognition groups, Black and Hispanic participants, as well as younger individuals with lower educational attainment, were less likely to have established a living will. Hispanic participants and younger individuals with lower educational attainment were also less likely to have a DPOAH and both a DPOAH and living will. Significant predictors of having a living will, DPOAH, or both that were not consistent among groups included rurality, marital status, and experiences of everyday discrimination. These findings underscore the importance of addressing disparities in advance care planning among older adults with diverse identities and levels of cognition. Understanding these differences is crucial for designing advance care planning for a diverse aging population facing health and cognitive decline.

